# GRN is a prognostic biomarker and correlated with immune infiltration in glioma: A study based on TCGA data

**DOI:** 10.3389/fonc.2023.1162983

**Published:** 2023-04-06

**Authors:** Su-Mei Xu, Hai-Yan Xiao, Zhong-Xu Hu, Xue-Feng Zhong, You-Jie Zeng, You-Xuan Wu, Dai Li, Tao Song

**Affiliations:** ^1^ Phase I Clinical Trial Center, Xiangya Hospital, Central South University, Changsha, China; ^2^ National Clinical Research Center for Geriatric Disorders, Xiangya Hospital, Central South University, Changsha, China; ^3^ Department of Neurosurgery, Xiangya Hospital, Central South University, Changsha, China; ^4^ Department of Anesthesiology, Third Xiangya Hospital, Central South University, Changsha, Hunan, China

**Keywords:** GRN, glioblastoma, biomarker, immune infiltration, prognosis, RNA-seq, bioinformatics analysis, prognostic signature

## Abstract

**Background:**

Among primary brain tumors, gliomas are associated with a poor prognosis and a median survival that varies depending on the tumor grade and subtype. As the most malignant form of glioma, glioblastoma (GBM) constitutes a significant health concern. Alteration in granulin(GRN) has been proved to be accountable for several diseases. However, the relationship between GRN and GBM remains unclear. We evaluated the role of GRN in GBM through The Cancer Genome Atlas (TCGA) database

**Methods:**

First, we assessed the relationship between GRN and GBM through the GEPIA database. Next, the relationship between GRN and GBM prognosis was analyzed by logistic regression and multivariate cox methods. Using CIBERSORT and the GEPIA correlation module, we also investigated the link between GRN and immune infiltrates in cancer. Using the TCGA data, a gene set enrichment analysis (GSEA) was performed. We also employed Tumor Immune Estimation Resource (TIMER) to examine the data set of GRN expression and immune infiltration level in GBM and investigate the cumulative survival in GBM. We also validated tissues from GBM patients by Western blotting, RT-qPCR, and immunohistochemistry.

**Results:**

Increased GRN expression was shown to have a significant relationship to tumor grade in a univariate study utilizing logistic regression. Furthermore, multivariate analysis disclosed that GRN expression down-regulation is an independent predictive factor for a favorable outcome. GRN expression level positively correlates with the number of CD4+ T cells, neutrophils, macrophages, and dendritic cells (DCs) that infiltrate a GBM. The GSEA also found that the high GRN expression phenotype pathway was enriched for genes involved in immune response molecular mediator production, lymphocyte-mediated immunity, cytokine-mediated signaling pathway, leukocyte proliferation, cell chemotaxis, and CD4+ alpha beta T cell activation. Differentially enriched pathways in the Kyoto Encyclopedia of Genes and Genomes (KEGG) include lysosome, apoptosis, primary immunodeficiency, chemokine signaling pathway, natural killer cell-mediated cytotoxicity, and B cell receptor signaling pathway. Validated result showed that GRN was upregulated in GBM tissues. These results suggested that GRN was a potential indicator for the status of GBM.

**Conclusion:**

GRN is a prognostic biomarker and correlated with immune infiltrates in GBM.

## 1 Introduction

Glioma, a typical malignant brain tumor, exhibits diffuse infiltration, no discernible boundaries, unlimited proliferation, and high invasion. Glioma are graded from I to IV by the World Health Organization (WHO) according to their histomorphology for diagnosis, treatment planning, and prognosis (1–3). Glioblastoma (GBM) is an aggressive type of glioma associated with resistance to treatments and recurrence; patients with GBM usually have a short survival rate (4). It is responsible for 50%-60% of all brain tumors and 12%-15% of all intracranial tumors (5, 6). About 75% of temozolomide (TMZ) treated GBM patients die within 2 years owing to recurrence, demonstrating that GBM patient survival rates remain dismally poor (7). Clinicians now have a pessimistic outlook on gliomas because of all the difficulties in treating them. Therefore, doctors and researchers dove headfirst into finding molecular markers associated with cancers to use with a patient’s pathological categorization, which ultimately determines the therapy that would be most effective for them (8, 9).

Numerous neurological illnesses may be brought on by mutations in the granulin (GRN) gene, and this variation shows an allele dose-dependent pattern (10). Microglia are resident innate immune cells of the CNS, and their number remains stable throughout life due to their ability to self-renew (11). Progranulin (encoded by GRN) is expressed in various tissues and organs throughout development and maturity, including epithelia, solid organs, immune cells, bone marrow, and the nervous system. Intracellular expression is higher in neurons and activated microglia in the brain (12). Its precursor protein (progranulin) is also known as teratoma cell-derived growth factor (13). Granulins are a family of protein growth factors involved in cell proliferation (14), and GRN family members play an essential role in normal development, wound healing, and tumorigenesis. GRNs may have cytokine-like activities and may play a role in inflammation, tissue remodeling, and wound repair. Loss of function mutations in the GRN gene is related to neurodegenerative and neurological diseases (15). According to Wang et al., GRN may be a prognostic factor for GBM and is likely implicated in the evolution of astrocytomas (16). GRN has emerged as a key prognostic marker for overall survival (OS) and disease-free survival (DFS) in patients with lower-grade glioma (LGG) and GBM (17).

Only a little data is known about the efficacy of GRN in gliomas; hence it has not been thoroughly tested in this context. TCGA is the source of our data. The association between GRN and GBM was also found using the R and GEPIA. Using the TIMER and a recently developed metagene method called CIBERSORT, we analyzed the number of tumor-infiltrating immune cells (TIICs) in various tumor microenvironments. We also used similar techniques to assess GRN’s relationship to immune cells infiltrating tumors. Consequently, we were able to add to the growing body of research on the potential benefits of GRN in GBM. The results also revealed a putative mechanism and association between GRN and tumor-immune interactions.

## 2 Materials and methods

### 2.1 Data acquisition

We retrieved gene expression profiles and clinical data from the TCGA (18) for 169 tumor tissues and 207 normal tissues from GBM patients. Subsequent processing eliminated records without adequate or missing age and overall survival time information. The initial step in converting RNA sequencing data to values comparable to those obtained from microarrays included transforming the raw count data. Tumor tissues were then separated into 2 groups based on the GRN expression to investigate the roles of GRN expression on the immunological microenvironment. According to the median cutoff, the survival and survminer packages were applied for Kaplan-Meier survival curve.

### 2.2 Survival and expression analysis by GEPIA

The relation between GRN expression and clinicopathologic data in GBM was verified using GEPIA (http://gepia.cancer-pku.cn/index.html). The GTEx and TCGA projects collected RNA sequencing expression data from 9,736 cancers and 8,587 normal samples, respectively; this data was then analyzed using the webserver GEPIA (19). Using GEPIA, we evaluated survival curves of differential GRN expression to see whether or not there was a link between gene expression and prognosis in GBM patients. At the same time, GRN expression was compared throughout pathological stages by means of a stage plot. In addition, differential expression of GRN was computed by plotting boxplots with disease status (Normal or Tumor) as the independent variable.

### 2.3 Using TIMER to produce an inference on the number of tumor-infiltrating immune cells

We used the TIMER 2.0 (https://cistrome.shinyapps.io/timer/) for systematically investigating immune infiltrates across several cancer types (20). Deconvolution, a statistical method that utilizes gene expression profiles to predict the amount of TIICs, is used by TIMER 2.0 (20). When estimating immune infiltrates, the TIMER database from The Cancer Genome Atlas was used, which contained 10,897 samples from 32 different cancer types. In this set of studies, we explored the GRN expression in several cancers and how it related to the presence of immune infiltrates. Using gene modules, the immune system responds with B cells, macrophages, CD4+ T cells, CD8+ T cells, neutrophils, and DCs, among other cell types in the infiltrate (21).

### 2.4 Gene set enrichment analysis

GSEA refers to a computer approach for assessing the statistical significance of a previously established collection of genes and the presence of concordant differences between two biological states (22, 23). GSEA was used to compile a preliminary list of gene classes based on their link with GRN expression in this research. Our findings of significant differences in survival between high- and low-GRN groups are further upon using this computational approach. Each study was conducted with a total of one thousand permutations of gene sets. We proposed using GRN expression as the phenotypic label. The nominal p-value and normalized enrichment score (NES) were also used to rank the enriched pathways in each phenotype (24). Significantly enriched gene sets were those with a nominal p-value and false discovery rate (FDR) of < 0.05.

### 2.5 Gauging the immune response of 22 TIICs in GBM *via* CIBERSORT

Gene expression deconvolution method CIBERSORT (25) (http://cibersort.stanford.edu/) can compare how one group of genes has changed in expression relative to the whole sample. As a result, this method provides a very accurate estimate of TIIC concentration. Cell heterogeneity research (26, 27) has received increased attention because of CIBERSORT’s reliable functioning. Using CIBERSORT, we quantified the immune response of 22 GBM TIICs to analyze their relationship to survival and molecular sub-population (28). In brief, standard annotation files were used to prepare gene expression datasets before they were uploaded to the CIBERSORT online platform and processed by the algorithm using the signature matrix’s default value of 1000 permutations. CIBERSORT used Monte Carlo sampling to obtain a P-value for deconvolution, providing a confidence interval for the findings. We utilized 160 TCGA samples, which have expression data for all genes, to evaluate the impact of GRN expression. We created a vioplot by dividing our set of 160 samples in half, assigning each half to either low or high expression.

### 2.6 Statistical analysis by R-4.2.1

R-4.2.1 was used to combine and analyze the TCGA data. Logistic regression was used to study the associations between the clinical data and GRN expression. The impact of GRN expression and other clinicopathological variables (gender and age) on survival was also assessed using Multivariate Cox analysis. The significance level of 0.05 was chosen as the significance threshold. We used the correlation heatmap, a visualization showing the relationship between each pair of immune cells in samples, to identify 22 distinct kinds of immune cell correlations.

### 2.7 Glioma tissue sample collection

Twenty-two glioma tissue samples were obtained from patients admitted to the Xiangya Hospital of Central South University between January 2008 and November 2021. There were ten male and 12 female patients aged 15 - 71 years old (median 47.1 years). Tumors were classified according to the 2016 WHO classification: 9 WHO II cases, 5 WHO III cases, and 8 WHO IV tumor cases. The patients had no history of radiotherapy or chemotherapy before surgery. All samples were analyzed by immunohistochemistry staining (IHC). Moreover, 14 pairs of glioma samples and normal brain samples were obtained and stored at −80°C for real-time quantitative polymerase chain reaction (RT-qPCR), and eight pairs of glioma samples and normal brain samples were obtained and stored at −80°C for Western Blot. The Ethics Committee of Xiangya Hospital of Center South University approved this study.

### 2.8 Western blot

Tissues were lysed in the RIPA buffer, and protein concentration was detected with the BCA protein assay kit (Beyotime, China). The 60-μg proteins were fractionated by SDS-PAGE on 10% SDS-acrylamide gel. The membrane was blocked with 5% skim milk for 1 h. After culturing with primary (GRN, ABclonal and α-tubulin, Servicebio) and secondary antibodies, the electro chemiluminescence kit (ECL; Solarbio, China) was used to detect the protein blots. The antibody information is listed in **Supplementary Table S1**.

### 2.9 Real-time quantitative polymerase chain reaction (RT-qPCR)

The extraction of total RNA, generation of cDNA, and RT-qPCR was achieved as described in the study (29). Gene expression was measured by 2-ΔCt, and the relative gene expression was assayed by 2-ΔΔCt according to the study (30). Primers used in this study were synthesized by Sangon Biotech (Shanghai, China) as the studies (29, 31), and the sequences are shown in **Supplementary Table S2**.

### 2.10 Immunohistochemistry

Human tissues were processed into formalin-fixed and paraffin-embedded specimens. Immunohistochemistry (IHC) was performed by means of streptavidin coupled with peroxidase. The samples were incubated overnight with GRN antibodies (1:100, Proteintech), followed by incubation with the biotinylated secondary antibody. Finally, the signals were detected using an Olympus BX41 microscope.

## 3 Results

### 3.1 Patient characteristics and multivariate analysis

**Figure 1:**
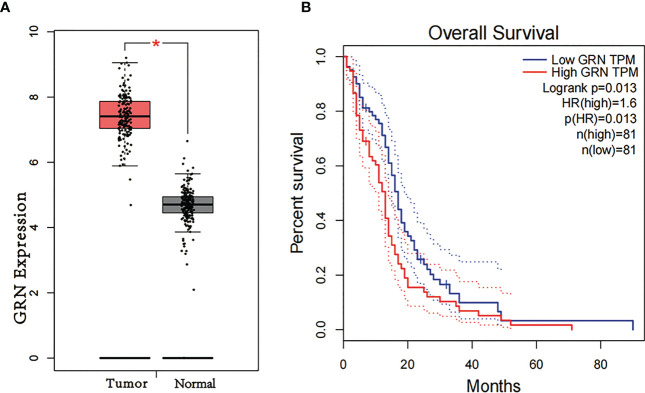
**(A)** Differential expression of GRN in different disease state (Tumor or Normal). **(B)** Survival curve of differential GRN expression were analyzed by GEPIA. *P < 0.05.

**Figure 2:**
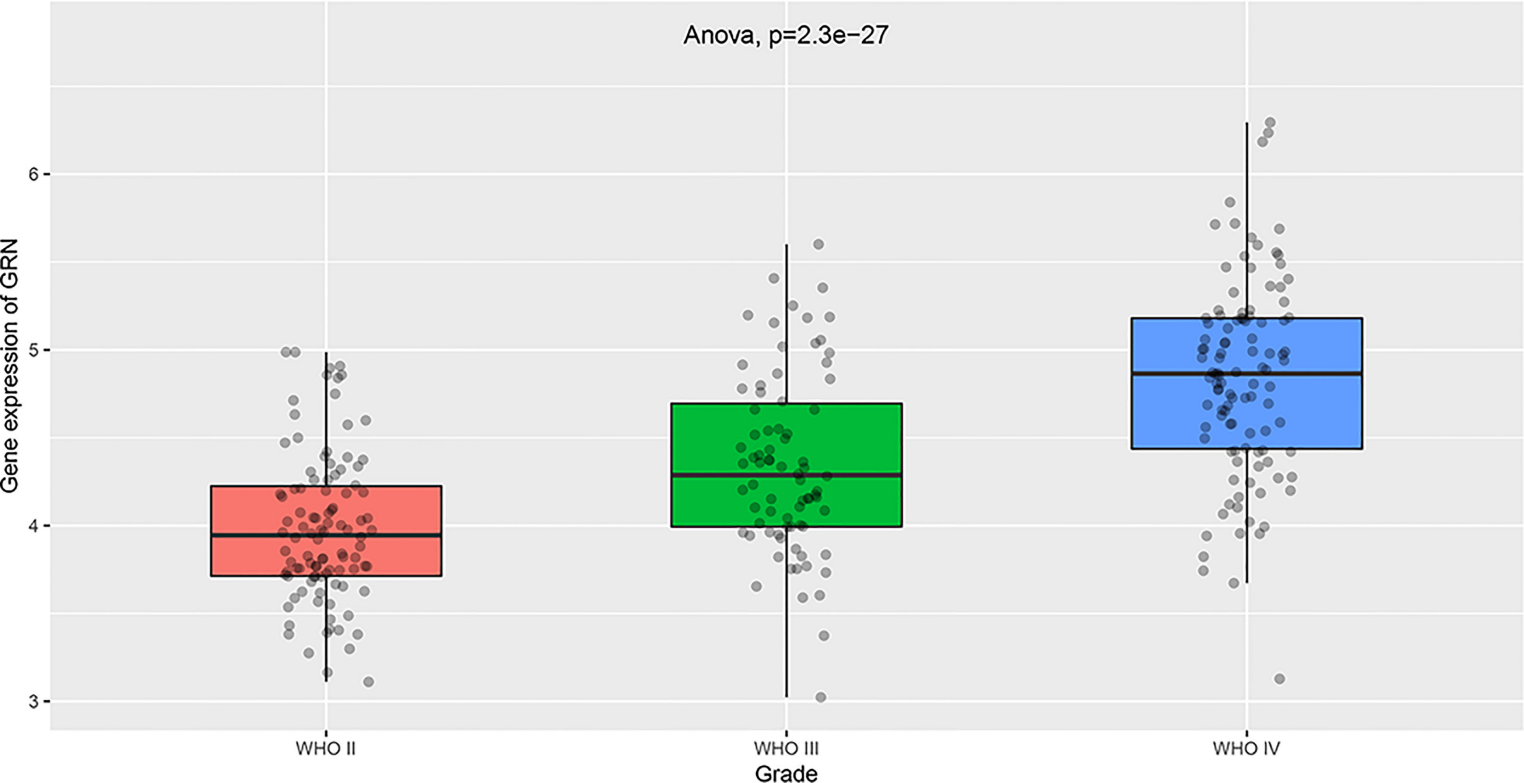
Differential expression of GRN in different cancer grade. (Based on the information of 103 G2 patients, 79 G3 patients and 139 G3 patients in TCGA database).

**Figure 3:**
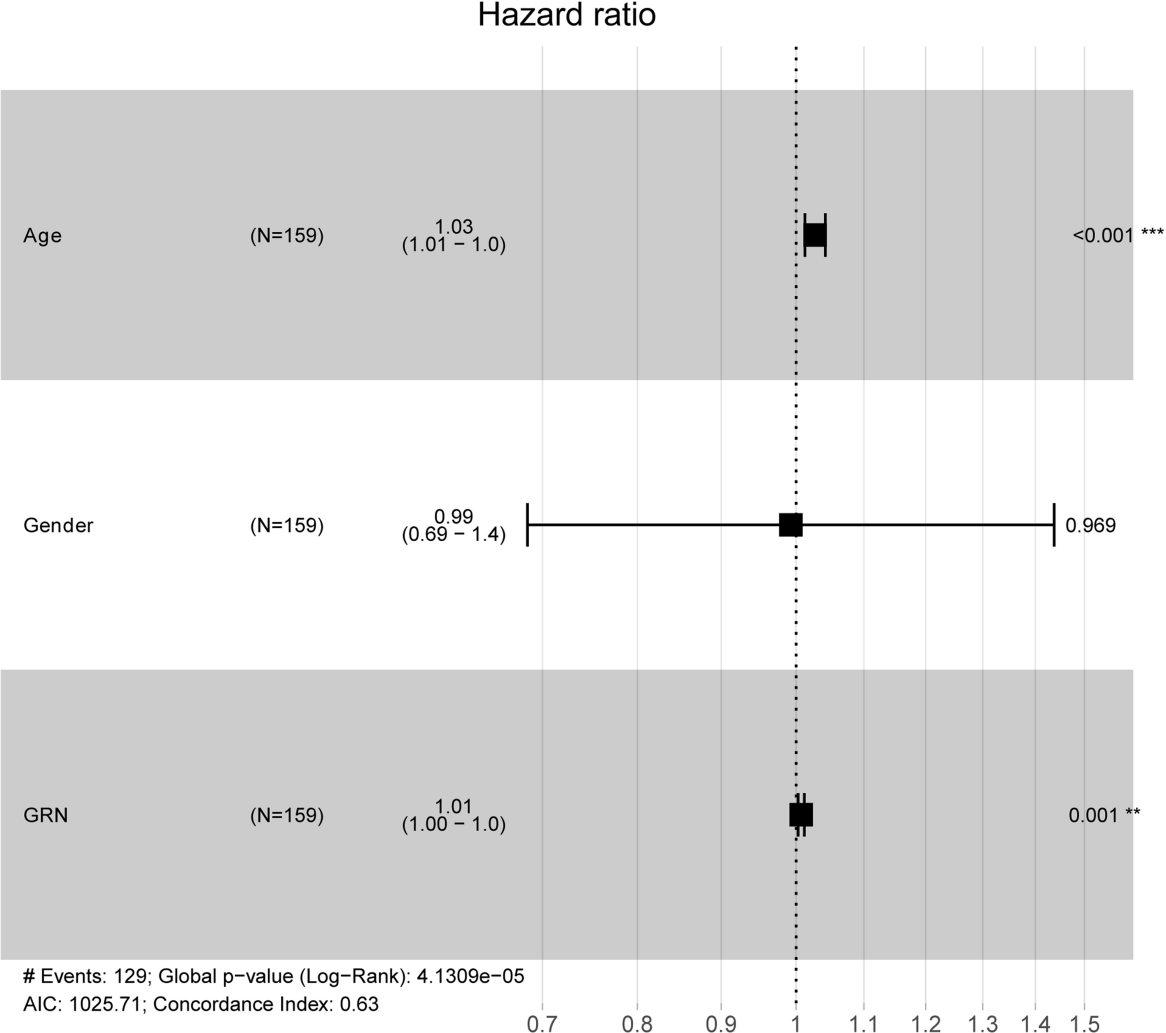
Multivariate Cox analysis of GRN expression and other clinical pathological factors. (As age and GRN expression are independent prognostic factors). **P < 0.01, ***P < 0.001.

To explore the association between GRN and GBM, we conducted an analysis using the GEPIA database. Our findings revealed that the expression level of GRN was significantly elevated in the GBM group relative to the control group (**Figure 1A**). Furthermore, survival analysis indicated that patients with high expression levels of GRN had a significantly shorter overall survival time compared to those with low expression levels (**Figure 1B**). Moreover, the results demonstrated a positive correlation between the expression levels of GRN and the grade of GBM, with higher GRN levels observed in more advanced stages (**Figure 2**). In addition, univariate and multivariate Cox risk regression analyses showed that GRN expression was an independent prognostic factor for OS (**Table 1**; **Figure 3**). Overall, the above findings suggest that GRN is closely related to GBM and is associated with poor prognosis of GBM.

**Table 1:** a. Association with overall survival and clinicopathologic characteristic in TCGA patients using Cox regression. b. Multivariate survival using Cox regression.

Clinical characteristics	HR(95%CI)	p-Value
a.		
age	1.026 (1.011-1.040)	0.000
gender	1.001 (0.693-1.446)	0.995
GRN	1.007 (1.002-1.011)	0.002
b.		
age	1.027 (1.012-1.041)	0.000
GRN	1.007 (1.003-1.012)	0.001

### 3.2 Immuno-infiltration analysis

**Figure 4:**
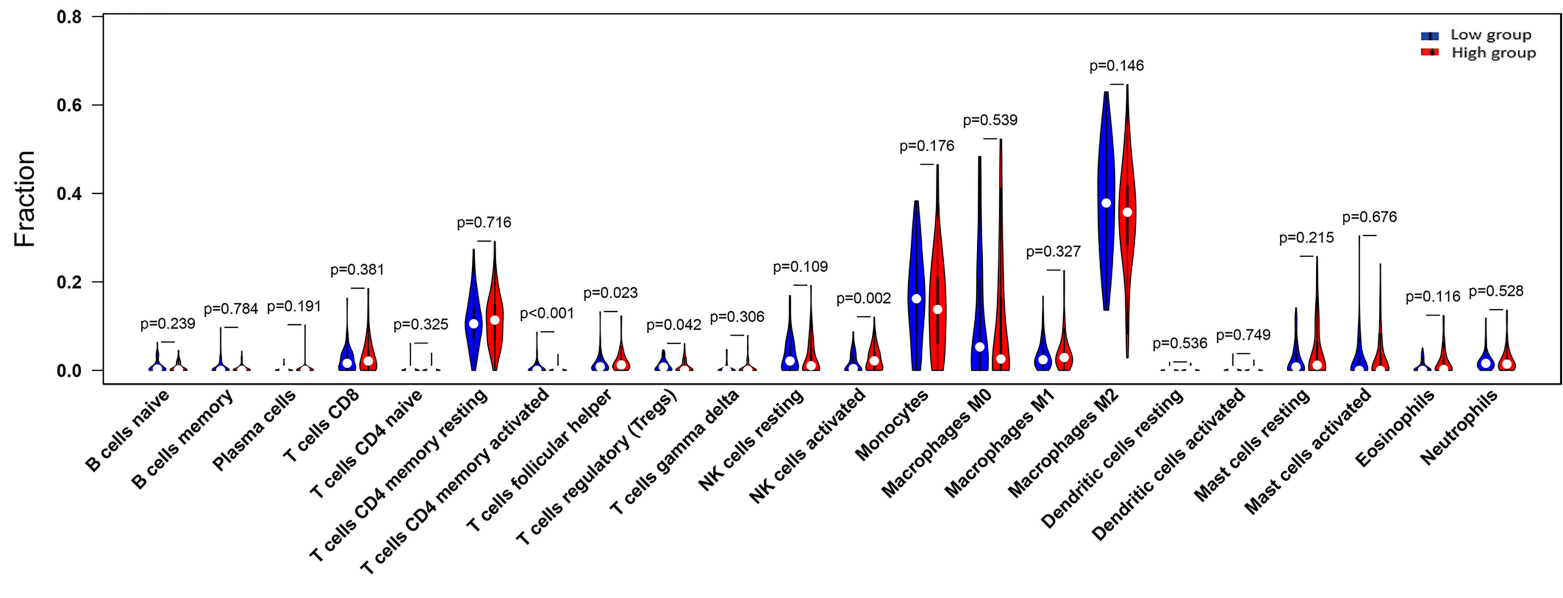
The proportion of 22 subpopulations of immune cells (T cells CD4 memory activated, T cells follicular helper, T cells regulatory (Tregs) and NK cells activated are main immune cells effected by GRN expression. Among them, T cells CD4 memory activated (p <0.001), T cells regulatory (Tregs) (p = 0.042) are decreased in high expression group compared with low expression group. In contrast, T cells follicular helper (p = 0.023), NK cells activated (p = 0.002) are increased in high expression group compared with low expression group; the blue is low GRN expression group and the red is high GRN expression group).

We performed immune infiltration analysis through the TIMER 2.0 database, thus exploring the potential association of GRN with immune cells in the GBM cohort. The 160 tumor samples were divided into GRN high expression group and GRN low expression group according to the median GRN expression level. **Figure 4** demonstrates the relative expression levels of 22 immune cells in the two groups. Specifically, in comparison to the GRN low-expression group, the GRN high-expression group exhibited lower levels of CD4 (+) memory T cells (p < 0.001) and Tregs (p = 0.042). Furthermore, the GRN high-expression group demonstrated a greater proportion of follicular helper T cells (p = 0.023) and activated NK cells (p = 0.002) than the GRN low-expression group.

**Figure 5:**
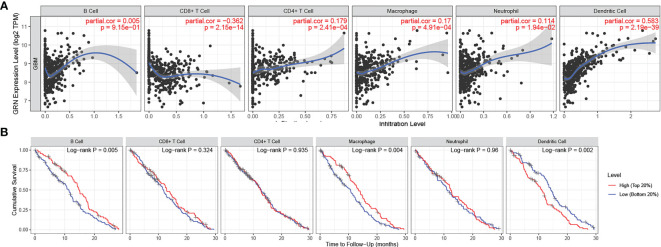
**(A)** GRN expression level has significant positive correlations with infiltrating levels of B cell, CD4+ T cells, Macrophages, Neutrophils and DCs in GBM. (A positive correlation exists between the GRN expression level and infiltrating levels of B cell(r = 0.005, P = 9.15e-01), CD4+ T cells (r = 0.179, P = 2.41e-04), Macrophages (r = 0.17, P = 4.91e-04), Neutrophils (r = 0.114, P = 1.94e-02), and DCs (r = 0.583, P = 2.19e-39) in GBM) **(B)** cumulative survival is related to B cell, Macrophages and DCs in GBM. (The B cell, Macrophages and DCs are factors related to the cumulative survival rate of GBM over time).

Subsequently, the correlation between GRN and multiple immune cells was calculated and the correlation between immune cells and GBM prognosis was identified through the TIMER 2.0 database (**Figures 5A, B**). Interestingly, macrophages and dendritic cells were associated with both GRN expression levels and GBM prognosis. Thus, there may be interactions between the two cell types and GRN that deserve future in-depth study.

### 3.3 Gene sets enriched in GRN expression phenotype

**Figure 6:**
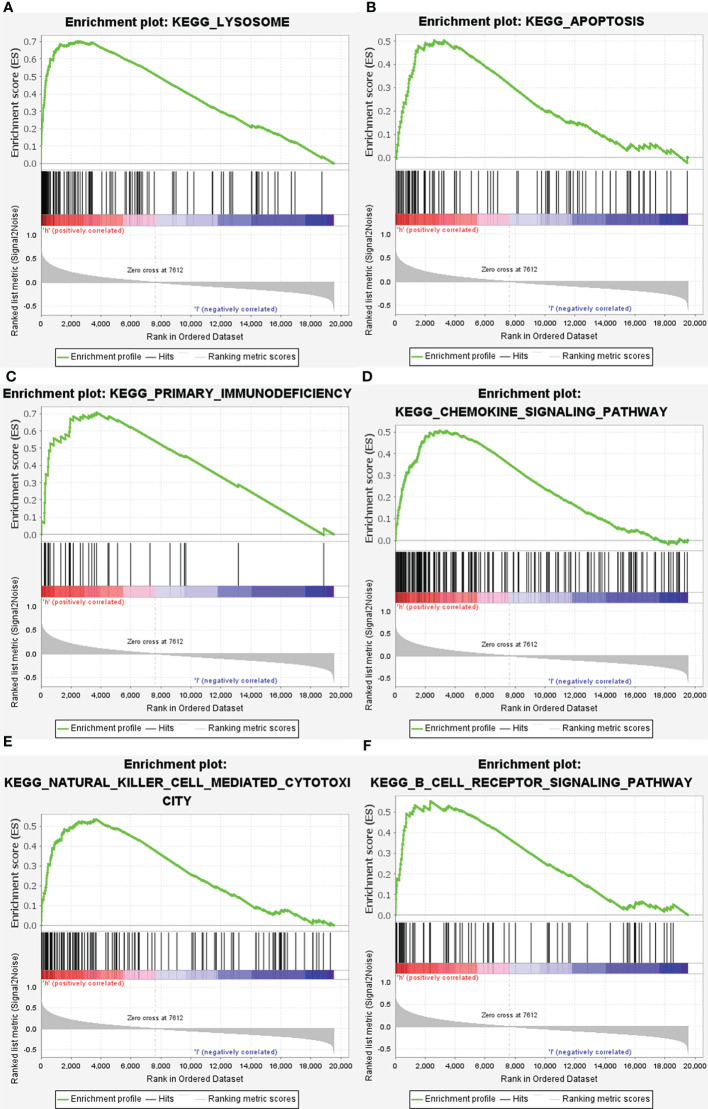
Enrichment plots from gene set enrichment analysis (GSEA). GSEA results showing differential enrichment of genes in KEGG with high GRN expression: lysosome **(A)**, apoptosis **(B)**, primary immunodeficiency **(C)**, chemolzine signaling pathway **(D)**, natural killer cell mediated cytotoxicity **(E)** and B cell receptor signaling pathway **(F)** were significantly differential enrichment based on NES, NOM P value, and FDR value.

**Figure 7:**
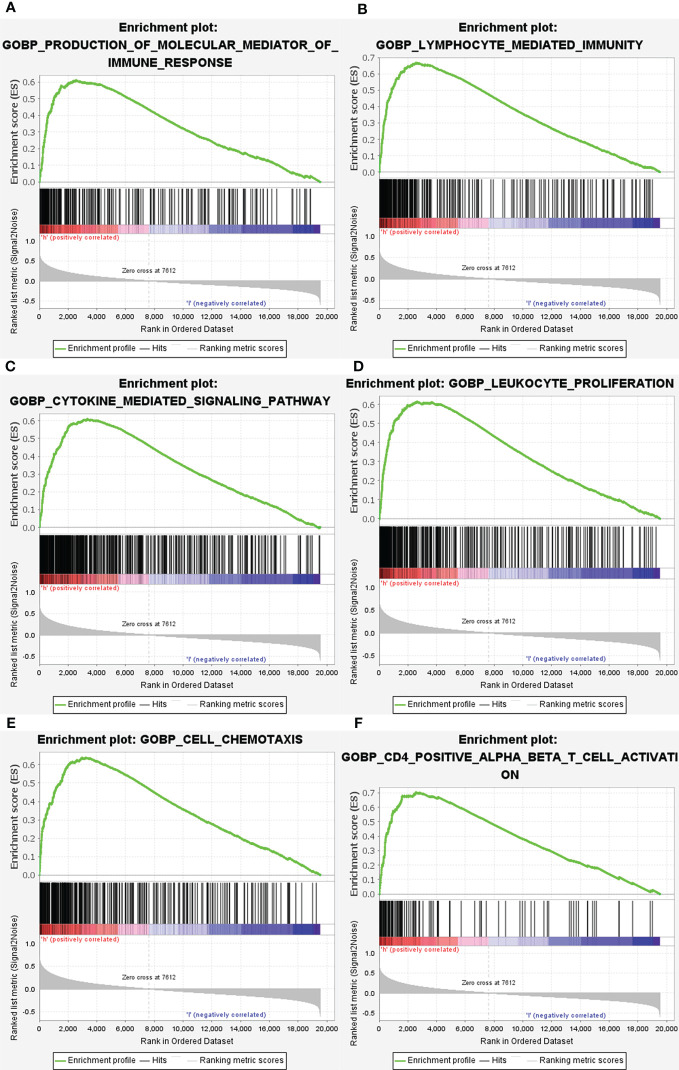
Enrichment plots from gene set enrichment analysis (GSEA). GSEA results showing differential enrichment of genes in GO with high GRN expression: production of molecular mediator of immune response **(A)**, lymphocyte mediated immunity **(B)**, cytokine mediated signaling pathway **(C)**, leukocyte proliferation **(D)**, cell chemotaxis **(E)** and CD4 positive alpha beta T cell activation **(F)** were showed significantly differential enrichment based on NES, NOM P value, and FDR value).

Subsequently, the altered pathways between the GRN high-expression and GRN low-expression groups were identified by GSEA. In term of KEGG, lysosome, apoptosis, primary immunodeficiency, chemokine signaling pathway, natural killer cell-mediated cytotoxicity, and the B cell receptor signaling pathway were significantly upregulated in the GRN high expression group (**Figure 6**). In addition, in the term of GO_BP, immune response molecular mediator production, lymphocyte-mediated immunity, cytokine-mediated signaling pathway, leukocyte proliferation, cell chemotaxis, and CD4+ alpha beta T cell activation were significantly upregulated in the GRN high expression group (**Figure 7**). **Table 2** shows the details of the enrichment results of the above terms. Overall, the above results suggest that GRN may impact the GBM progression through these terms.

**Table 2:** Gene sets enriched in phenotype.

Gene set name	NES	NOM p-val	FDR q-val
KEGG_LYSOSOME	2.383798	0.0	0.0
KEGG_APOPTOSIS	1.907874	0.0	0.011608
KEGG_PRIMARY_IMMUNODEFICIENCY	1.981136	0.004040	0.006105
KEGG_CHEMOKINE_SIGNALING_PATHWAY	1.864272	0.002145	0.015102
KEGG_NATURAL_KILLER_CELL_MEDIATED_CYTOTOXICITY	1.933129	0.0	0.009922
KEGG_B_CELL_RECEPTOR_SIGNALING_PATHWAY	1.892072	0.004	0.013
GO_PRODUCTION_OF_MOLECULAR_MEDIATOR_OF_IMMUNE_RESPONSE	2.280836	0.0	2.9787753E-4
GO_LYMPHOCYTE_MEDIATED_IMMUNITY	2.302876	0.0	2.3400079E-4
GO_CYTOKINE_MEDIATED_SIGNALING_PATHWAY	2.291129	0.0	2.6831732E-4
GO_LEUKOCYTE_PROLIFERATION	2.238176	0.0	5.901053E-4
GO_CELL_CHEMOTAXIS	2.226881	0.0	5.822444E-4
GO_CD4_POSITIVE_ALPHA_BETA_T_CELL_ACTIVATION	2.233316	0.0	6.378307E-4

### 3.4 Validation of GRN expression in GBM Tissue

**Figure 8:**
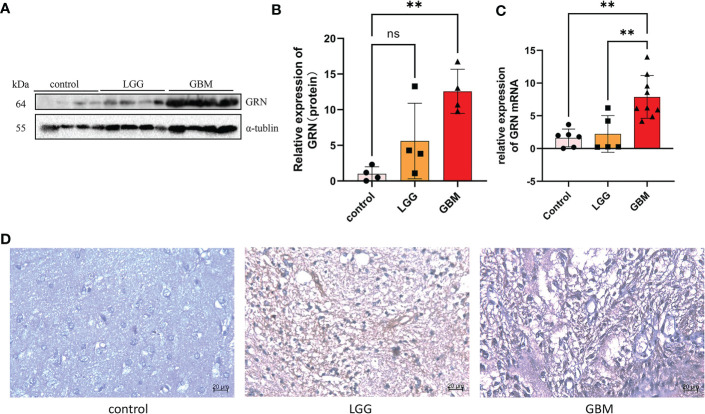
GRN is upregulated in GBM tissues. **(A, B)** Western blot was used to detect the expression of GRN in control and tumor of GBM patients (n = 4). **(C)** RTqPCR analysis of the different GRN expressions in gliomas and adjacent normal tissues. **(D)** IHC staining of GRN protein expression in gliomas and adjacent normal tissues. **p< 0.01. Scale bar, 20µm. ns, not significant.

The result of the Western blot of 12 GBM patients showed that the expression of GRN in high-grade GBM tissues was significantly higher than that in the control group and the low-grade group (**Figures 8A, B**). To further verify the GRN expression from the mRNA level, RT-qPCR was also conducted based on patient tissues and further verified the significant differences of GRN expression between tumor and normal (**Figure 8C**). Then, we performed immunohistochemical experiments on the tissues to detect the distribution and expression of GRN. We found that the tumor had higher GRN expression than normal (**Figure 8D**).

## 4 Discussion

Secreted GRN is expressed in several CNS cell types and shows evolutionary conservation (32). First identified as a growth factor with roles in cell division, tissue repair, tumor formation, and inflammatory processes, its discovery was a game changer (33, 34). Cancers of the skin, breast, astrocytoma, lung, ovary, uterus, head and neck, liver, lymphoma, multiple myeloma, and leukemia all have GRN as a tumor promoter. There seems to be a dearth of information on the prognostic value of GRN in GBM, at least in the literature we have accessed. Consequently, we investigated GRN’s possible significance in GBM and performed the first comprehensive investigation of GRN expression in a large group of human GBM patients. We analyzed 321 glioma patients retrospectively using a combination of RNA-seq and clinical data, with the results confirmed by histology. We found that the GRN mRNA expression level correlates with glioma patients’ tumor grade.

Here, we document a correlation between the degree of GRN expression and the prognosis of GBM. Reduced expression of GRN is related to a better prognosis in GBM patients. One of the independent prognostic variables for a good outcome is a decrease in GRN expression. Our research further shows that GRN expression is linked to the presence of a wide variety of immune marker sets and degrees of immune infiltration in GBM. As a result, we hypothesized that GRN could have a role in tumor immunology.

Further, it shows promise as a biomarker for cancer. Using the online database GEPIA, we determine whether there is a link between GRN expression and survival in GBM patients. Positive outcomes are linked to GRN expression being lowered. GRN expression was also shown to vary across GBM normal and malignant tissues. We use TCGA data to learn more about the context and causes of GRN expression in cancer. GRN expression was shown to be correlated with tumor stages in TCGA analysis using R-4.2.1. Expression of GRN was a significant independent prognostic factor in GBM patients using multivariate analysis. Key findings from our research include a relationship between GRN expression and immune infiltration levels in GBM. Activated natural killer (NK) cell and T follicular helper (Tfh) cell infiltration levels are positively correlated with GRN expression in GBM, as determined by CIBERSORT. Similar associations have been found between GRN expression and various immune cell markers, suggesting a pivotal role of GRN in controlling the GBM tumor immunological microenvironment.

Many studies have focused on T-cells when analyzing the function of TIICs in human malignancies, and many have reported on the cells’ response to immune checkpoint suppression and their survival (35, 36). This research adds to the growing body of work that has established T-cells as a protective factor in prognosis (37). It is well recognized that B-cells play an important role in solid malignancies, with CD20 expression correlating to improved overall survival in breast and ovarian cancer (38). T-cell and B-cell gene expressions, particularly B-cell receptor (BCR) segment expression, were shown to be substantially associated with previous research (39, 40). Strong correlations were found between the expression of immunological signatures on various kinds of immune cells, indicating the presence of a heterogeneous but somewhat predictable and persistent tumor-immune infiltration bolstered by B-cells (41, 42).

Through the use of GSEA and TCGA data, we were able to delve further into the roles of GRN in GBM. GSEA showed that lysosome, primary immunodeficiency, chemokine signaling pathway, natural killer cell-mediated cytotoxicity, apoptosis, and B cell receptor signaling pathway in KEGG and production of molecular mediator of the immune response, lymphocyte-mediated immunity, cytokine-mediated signaling pathway, leukocyte proliferation, cell chemotaxis, and CD4+ alpha beta T cell activation in GO are differentially enriched in GRN high expression phenotype. Together, these data revealed that GRN has promise as a therapeutic target and prognostic marker in GBM.

GRN expression was also positively correlated with the number of CD4+ T cells (r = 0.179, P = 2.41e-04), macrophages (r = 0.17, P = 4.91e-04), neutrophils (r = 0.114, P = 1.94e-02), and DCs (r = 0.583, P = 2.19e-39) infiltrating GBM. Collectively, our results highlighted the profound role that GRN plays in the GBM immune infiltration, B cells, macrophages, and DCs cells.

## 5 Conclusion

We conclude that the poor survival in GBM has a probable prognostic molecular marker known as the GRN expression. Finally, the results further suggest that for improved clinical outcomes among GBM patients, we could use an independent prognostic factor known as the low GRN mRNA expression. We strongly recommend further research on the subject matter to progressively improve the evidence on the biological impact of GRN.

## Data availability statement

The original contributions presented in the study are included in the article/**Supplementary Material**. Further inquiries can be directed to the corresponding authors.

## Ethics statement

The studies involving human participants were reviewed and approved by Medical Ethics Committee of Xiangya Hospital, Central South University. The patients/participants provided their written informed consent to participate in this study.

## Author contributions

DL and TS conceptualized and designed the study. S-MX and H-YX participated in the bioinformatics analyses. S-MX drafted the manuscript. Z-XH, Y-JZ, X-FZ, and Y-XW participated in the design of the study. S-MX and H-YX helped to revise the study. All authors contributed to the article and approved the submitted version.
