# Supplementary material for: GRN is a prognostic biomarker and correlated with immune infiltration in glioma: A study based on TCGA data
Source: Front Oncol. 2023 Apr 6;13:1162983. doi: 10.3389/fonc.2023.1162983 (PMC10117795; doi:10.3389/fonc.2023.1162983)
Supplement: Supplementary file 1 [file DataSheet_1.pdf]

Supplementary Table S1. The antibody used in study

Antibody	Species reactivity	Host species	Supplier	ConcentRion
Anti-GRN	Human	Rabbit	ABclonal	1：1000
Anti-α-tubulin	Human	Rabbit	Servicebio	1：5000
Goat Anti-Rabbit IgG H&L (HRP)	Rabbit	Goat	Signalway Antibody	1：5000

Supplementary Table S2. Sequences of the primers used to quantitate gene expression.

Gene	Forward primer (5′-3′)	Reverse primer (5′-3′)
GRN	ATCTTTACCGTCTCAGGGACTT	CCATCGACCATAACACAGCAC
GAPDH	CTCCTGCACCACCAACTGCT	GGGCCATCCACAGTCTTCTG
